# Acting like a Tough Guy: Violent-Sexist Video Games, Identification with Game Characters, Masculine Beliefs, & Empathy for Female Violence Victims

**DOI:** 10.1371/journal.pone.0152121

**Published:** 2016-04-13

**Authors:** Alessandro Gabbiadini, Paolo Riva, Luca Andrighetto, Chiara Volpato, Brad J. Bushman

**Affiliations:** 1 University of Milano Bicocca, Department of Psychology, Milan, Italy; 2 University of Genova, Department of Education, Genova, Italy; 3 The Ohio State University, School of Communication, Columbus, Ohio, United States of America; 4 VU University Amsterdam, Amsterdam, The Netherlands; University of Toledo, UNITED STATES

## Abstract

Empathy—putting oneself in another’s shoes—has been described as the “social glue” that holds society together. This study investigates how exposure to sexist video games can decrease empathy for female violence victims. We hypothesized that playing violent-sexist video games would increase endorsement of masculine beliefs, especially among participants who highly identify with dominant and aggressive male game characters. We also hypothesized that the endorsement of masculine beliefs would reduce empathy toward female violence victims. Participants (*N* = 154) were randomly assigned to play a violent-sexist game, a violent-only game, or a non-violent game. After gameplay, measures of identification with the game character, traditional masculine beliefs, and empathy for female violence victims were assessed. We found that participants’ gender and their identification with the violent male video game character moderated the effects of the exposure to sexist-violent video games on masculine beliefs. Our results supported the prediction that playing violent-sexist video games increases masculine beliefs, which occurred for male (but not female) participants who were highly identified with the game character. Masculine beliefs, in turn, negatively predicted empathic feelings for female violence victims. Overall, our study shows who is most affected by the exposure to sexist-violent video games, and why the effects occur. (200 words)

## Introduction

“Empathy is about standing in someone else's shoes, feeling with his or her heart, seeing with his or her eyes. Not only is empathy hard to outsource and automate, but it makes the world a better place.”

—Daniel H. Pink, author

Empathy is an emotional response that corresponds to the feelings of another person, such as feeling distress when seeing another person in distress. Empathy does indeed make the world “a better place” to live and it is one of the best predictors of prosocial behavior [1,2,3]. Numerous studies have shown that playing violent video games reduces feelings of empathy and makes people numb to the pain and suffering of others (for a meta-analytic review see [4]).

People feel empathy for other individuals, not for objects. In some video games, such as the very popular *Grand Theft Auto* (*GTA*) games, female characters are treated as sex objects rather than as individuals worthy of respect. *GTA* main male characters are always depicted as hyper-masculine, dominant, and aggressive men. In contrast, the female characters are portrayed as sexual objects—usually prostitutes or pole-dancers—who are peripheral to the game narrative and whose sole purpose is to entertain the main male characters [5]. For example, after paying a prostitute for sex, players can kill her and get their money back. Rather than being punished for such behaviors, players are often rewarded (e.g., through points, extra health to their character, etc.).

Although sex-typed video game characters in the virtual world might affect perceptions of men and women in the real world, there is a dearth of research on this topic. In one study, male college students who saw photos of sex-typed male and female video game characters (vs. professional men and women) were more tolerant of sexual harassment against a female college student by a male professor [6,7] In another study, female college students who embodied sexualized video game characters (vs. non-sexualized game characters) were more likely to view themselves as a sexual object, which in turn increased their acceptance of rape myths [5]. In a third study, both male and female participants were more aggressive after playing a violent game as male character than as a female character [8]. Yet, none of these studies assessed the role of individual differences (i.e., moderators) and/or underlying mechanisms (i.e., mediators) of the obtained effects. The present research fills these important gaps in the literature by testing a moderator variable (i.e., identification with the violent-sexist video game character and gender), and a mediator variable (i.e., masculine beliefs) of the effects of exposure to violent sexist video games on empathy for female violence victims

## *Who* is Most Affected: The Moderating Role of Identification with the Game Character

Previous research has largely ignored moderators of the effects of violent-sexist video games on players. The notion of identification with a virtual avatar, as an online self-representation, has been investigated in past research [9,10,11]. In particular, some works have shown that when experiencing a virtual world, players are likely to establish a connection between themselves and their game character, and even imagine themselves to *be* that character [12,13,11]. For example, participants in one study [13] played either a first-person shooter war game or a racing game and then completed a measure of automatic attitudes using the Implicit Association Test [14]. The researchers found that participants who played a war game had stronger associations between military-related concepts and the self, whereas participants who played a racing game had stronger associations between racing-related concepts and the self. These results suggest an automatic shift in players’ implicit self-perceptions. Identification with violent video game characters can also influence behavior. For example, one study found that the more boys identified with violent game characters, the more aggressive they were after the game was turned off [11].

Building on these previous findings, in the present work we tested whether the identification with the game character would interact with exposure to violent sexist games in predicting a reduction in empathic feelings toward female violence victims. More specifically, we expected that players who highly identified with violent sexist game character would display a greater endorsement of masculine beliefs. Furthermore, we expected that the interactive effects of exposure to violent sexist games and identification with the game character on masculine beliefs would be stronger for male (compared to female) players. Drawing from this theoretical framework, we propose that identification with the game character and participants’ gender could play a key-moderating role in the effects of violent-sexist games on empathy for female violence victims.

## *Why* the Effect Might Occur: The Mediating Role of Masculine Beliefs

Previous research has largely ignored mediators of the effects of violent-sexist video games on players. One scholar notes that the video game culture assumes that the default player is male, which can lead to the maintenance of masculinity in the virtual world [15]. Another scholar proposed that adolescent and young adult males often use video game spaces to explore their masculine identity [16]. Masculinity refers to normative beliefs about how men are expected to think, feel, and behave [17]. Traditionally, men are considered to be aggressive, dominant, competitive, strong, powerful, and independent [17,18]. Theories of hegemonic masculinity assert that modern media convey myths about male dominance and female submission in order to support a patriarchal social structure [19]. In other words, media stereotypes construct a stylized view of masculinity and femininity that influences the thoughts, feelings, and behaviors of those who consume these media. In the mediated world, men are expected to control their feelings in order to be less vulnerable and more powerful. Emotions such as fear and empathy are prohibited because “real men” are not supposed to express these feelings [20,18]. However, not all emotions are prohibited. Feelings of anger and rage are encouraged in “real men” because they are associated with high status and power.

The portrayal of men in the media as socially powerful and physically violent reinforces assumptions about how men and boys should act in society, as well as how they should treat women and girls [21]. Exposure to sex-typed media characters can have real world consequence. For example, one study found that television programs that depict women as sex objects increased the likelihood of sexual harrassment [22]. A meta-analytic review found that masculine beliefs were positively associated with aggression against women [23]. In general, males are more likely than females to agree with myths and beliefs supportive of violence against women, show less empathy for female violence victims, and consider violence against females to be a less serious problem (see [24]). We propose that the exposure to violent and sexist video games could reinforce masculine beliefs, hampering the ability for players to feel empathy for female violence victims. Because masculine norms are strongly reinforced in *GTA* video games, we propose that *GTA* gameplay will increase masculine beliefs. Masculine beliefs, in turn, are expected to be negatively related to empathy for female violence victims.

## Overview

**Fig 1:**
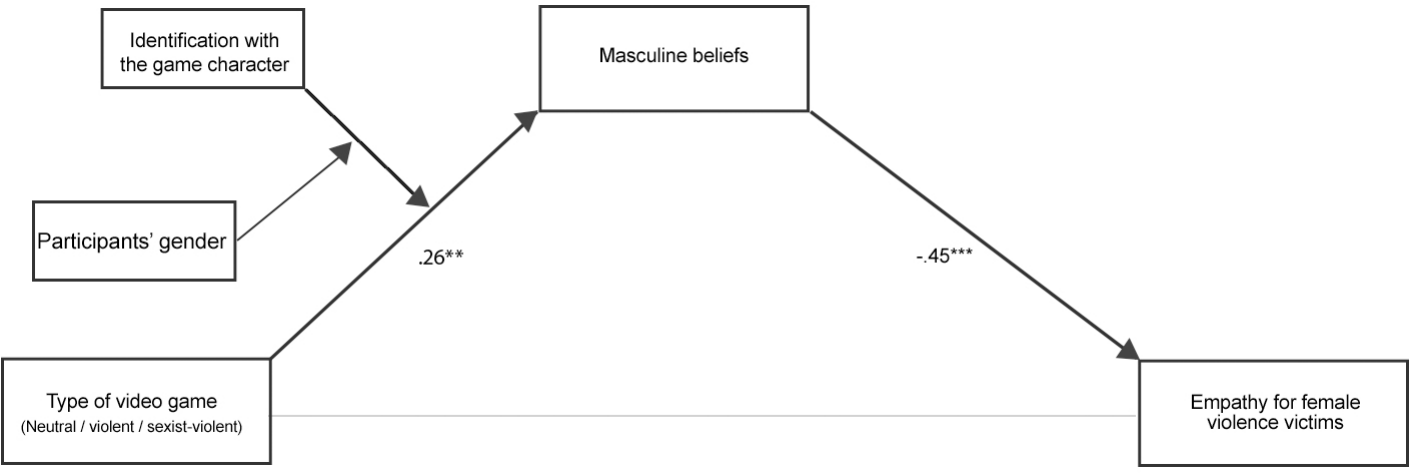
Conditional process moderated mediation model (Model 11 in PROCESS [24]).

The present study investigates the short-term effects of playing violent-sexist video games on empathy for female violence victims. We consider identification with the game character as one possible moderator, expecting larger effects of the game content for participants who strongly identify with the hyper-masculine, violent, male game character in *GTA*. Further, we proposed participants’ gender as second possible moderator, assuming that the effects of the game content would be stronger for male participants than for female participants. As a possible mediator, we propose that violent-sexist games reduce empathy for female violence victims by increasing masculine beliefs. Combining these hypotheses, we propose a conditional process model ([25]; see Fig 1). In this model, we predict that violent-sexist video games will increase masculine beliefs, which in turn will be negatively related to empathic feelings for female violence victims, especially among male participants who display higher levels of identification with the violent male game character. To test whether these effects are specific to violent-sexist video games, we also included violent-only video games in our design, as well as nonviolent (control) video games.

## Materials and Method

### Participants

Participants were 154 Italian high school student volunteers (43.4% male, 15 to 20 years old, *(M* = 16.82, *SD* = 1.24). The study was reviewed and approved by University of Milano-Bicocca ethics committee (Prot. N. 0024403/12) before the study began. Both the high school and the university review board gave their written consent for the study; parents' consent was collected through an official written communication sent by the office school management before the beginning of the data collection. Parental informed consent and participant written assent rates were both 100%.

### Procedure

Participants were told that investigators were testing cognitive abilities in order to develop a new video game that would be distributed in the near future. After providing basic demographic information (age, nationality, gender), participants were randomly assigned to play a violent-sexist video game (i.e., *GTA San Andreas* or *GTA Vice City*; both rated 18+ for violent and sexual content), a violent-only video game (i.e., *Half Life 1* or *Half Life 2*; both rated 16+ for violent action, but no sexual content or violence toward women), or a non-violent video game (i.e., *Dream Pinball 3D* or *Q*.*U*.*B*.*E*. *2*; both rated 10+ with no violent or sexual content).

*Half-Life* is a first-person shooter video game where the player has the same visual perspective as the character. In both games used in this study (*Half Life 1*, *Half Life 2*), players fight in a post-apocalyptic future. Although there is a female co-protagonist (Alyx), she is portrayed in a non-sexual manner. In contrast, all female characters in *GTA* are portrayed in a sexual manner. By selecting video games that use different representation of women, it is possible to differentiate between sexual violence exposure (i.e., *GTA*) and exposure to violent games not involving sexism exposure (i.e., *Half Life*).

*Dream Pinball 3D* is a classic pinball simulation game featuring different tables to play on, while *Q*.*U*.*B*.*E*. *2* is a first-person puzzler in which the player has to solve an array of physics-based challenges.

For all the three video game conditions, participants first watched the introductory video of the game for about one minute, in order to familiarize themselves with the game. Next, they practiced by playing a preselected scene for 5 minutes. During the practice session, participants were taught how to control their character and how to interact with the game world. When the practice session ended, participants played the game alone for 25 minutes

For all the three games, participants were asked to pursue a specific goal or mission. We attempted to keep the mission as similar as possible for the violent-sexist and the violent-only games. For violent-sexist games (both *GTA San Andreas* and *GTA Vice City*), the mission was to destroy a rival criminal gang. Both missions started in a private-club, and then move through the streets of the city. During the gameplay, players were frequently exposed to female prostitutes and lap dancers; indeed, those are common elements in all the episodes of the GTA saga. For the violent-only games (both *Half Life 1* and *Half Life 2*), players were asked to complete a mission whose goal was to destroy a group of enemies. The game’s action in both Half life 1 and Half life 2, takes place in a suburban area of the city, and then moves to a few abandoned buildings. For the neutral control games (both *Dream Pinball 3D* and *Q*.*U*.*B*.*E*. *2*), the mission was simply to accumulate as many points as possible.

All games in all conditions were played at an intermediate level to avoid boredom (from the game being too easy) and frustration (from the game being too difficult).

After gameplay, participants completed some video game manipulation checks. They were first asked to report the title of the video game they played, rated how violent, involving, and exciting they thought the game was (1 = *not at all* to 7 = *extremely*), and rated how sexualized the female figures in the game were (1 = *not at all* to 7 = *extremely*). Given the popularity of the GTA series among young people, we also measured how frequently they played the video game they were randomly assigned to play in this experiment (0 = *never played before* to 7 = *every day*).

One moderator was participant gender. As a possible second moderator, we measured how much participants identified with their video game character using the 6-item (e.g., “When I am playing, it feels as if I am my character” and “My character is an extension of myself”; 1 = *completely disagree* to 7 = *completely agree*) of the Player Identification Scale [26] (Cronbach α = .92).

As a possible mediator, we measured masculine beliefs. We used 12 items (e.g., “Boys should be encouraged to find a means of demonstrating physical prowess” and “It is OK for a guy to use any and all means to ‘convince’ a girl to have sex”; 1 = *completely disagree* to 7 = *completely agree*) from the revised Male Role Norms Inventory (MRNI-R; Cronbach α = .78) [27] Levant, Rankin, Williams, Hasan, & Smalley, 2010).

The dependent variable was how much empathy participants felt toward female violence victims. When people become desensitized to violence, they become numb to the pain and suffering of violence victims [28]. Participants were shown one of two photos (randomly determined) of an adolescent girl who had been physically beaten by an adolescent boy (see Figure A and Figure B in S1 Appendix). Participants rated whether they felt sympathetic, moved, compassionate, tender, warm, softhearted, disregarded (reverse-coded) and indifferent (reverse-coded) (see [29]) for her (1 = *not at all* to 7 = *very much*; Cronbach α = .83).

About 2 weeks later, following the completion of the study, all participants were fully debriefed. No participants expressed suspicion about the true purpose of the study. In particular, none of the participants reported a link between video games and gender-based violence or sexism. The experimenter then disclosed the purpose of the study, and discussed the potentially harmful short-terms effects of violent and sexist video games on players. A group discussion followed.

## Results

### Preliminary Results

#### Stimulus sampling

To increase the generalizability of our findings, we used two video games of each type and two photos of interpersonal violence [30]. Independent-sample *t*-tests found no significant differences between the two different violent-sexist games, between the two different violent-only games, or between the two nonviolent games on identification with the main video game character, masculine beliefs, or empathy for female violence victims (*ps*>.12). Thus, the two violent-sexist games were combined, the two violent-only games were combined, and the two nonviolent games were combined for subsequent analyses.

There were no significant differences between the two photos of the adolescent boy beating the adolescent girl on how much empathy participants felt for her (*p*s>.10). Thus, the data from two photos were combined for subsequent analyses.

#### Video game manipulation check items

Five items were included to assess whether the video game manipulation was successful. First, we checked the name of the game reported by each participant. All participants correctly named the video game they played. We then tested whether the violent video games were rated to be more violent than the nonviolent games. A one-way between subjects ANOVA found a significant difference in violence ratings for the video games, *F*(2,150) = 182.16, *p* < .001, *η*^2^ = .70. Post-hoc comparisons using Tukey’s Honestly Significant Difference (HSD) test indicated that the violent-sexist games (*M =* 5.11, *SD* = 1.23) and the violent-only games (*M =* 4.09, *SD* = 1.25) had significantly higher violence ratings than the nonviolent games (*M =* 1.20, *SD* = .53), *d*s = 4.13 and 3.01, respectively. However, the violent-sexist games also had higher violence ratings than the violence-only games (*M =* 5.11, *SD* = 1.23; *d* = .82). Then, we checked ratings of familiarity. A one-way between-subjects ANOVA found a significant difference in frequency of play for the video games, *F*(2,152) = 4.28, *p* = .015, *η*^2^ = .053. Post-hoc test revealed significantly that violent-sexist games had higher frequency of play (*M* = 2.10, *SD* = 1.54; *p* = .013) than both violent-only (*M* = 1.36, *SD* = 1.31) and neutral games (*M* = 1.58, *SD* = 1.03). Thus, violence ratings and frequency of play were included as covariates in all analyses. No statistically significant differences between violent-sexist (*M =* 4.18, *SD* = 1.56), violent-only (*M =* 3.94, *SD* = 1.42), and neutral games (*M =* 3.82, *SD* = 1.76) were found for game involvement, *F*(2,151) = 0.70, *p*>.49. Moreover, no significant differences between violent-sexist (*M =* 3.71, *SD* = 1.55), violent-only games (*M =* 3.65, *SD* = 1.68), and neutral games (*M =* 3.18, *SD* = 1.58) were found of game excitement, *F*(2,151) = 1.67, *p*>.19. Finally, we tested whether female characters were rated as more sexualized in the violent-sexist video games than in the violent-only games. As expected, female characters in the *GTA* games were rated to be more sexualized (*M =* 5.88, *SD* = 1.43) than female characters in the *Half Life* games (*M =* 2.16, *SD* = 1.31), *t*(101) = 13.67, *p* < .001, *d* = 2.72. Taken together, these results suggest that the video game manipulation was successful.

### Primary Results

Since playing violent video games that are not necessarily sexist have been already shown to reduce feelings of empathy, we contrast coded the type of video game as follow: +1 = violent-sexist games, 0 = violent-only games, -1 = non-violent games. Data analysis revealed that the type of video game was positively associated with masculine beliefs (*r* = .203, *p* = .011). Participant gender (1 = male vs. 0 = female) was associated with both masculine beliefs (*r* = .507, *p* < .001) and identification with the game character (*r* = .241, *p* = .003). Mean comparison revealed that participant gender impacted the masculine beliefs such that male participants showed greater endorsement of masculine beliefs (*M* = 3.23, *SD* = 0.82) than female did (*M* = 2.44, *SD* = 0.52; *t*(152) = 7.25; *p* < .001; *d* = 1.15). Participant gender also influenced the identification with the game character such that boys identified more with the game character (*M* = 4.63, *SD* = 1.20) than girls (*M* = 3.96, *SD* = 1.45; *t*(152) = 3.05; *p* = .003; *d* = 0.50). Further, the level of identification with the game character was positively associated with masculine beliefs (*r* = .197, *p* = .014). Finally, masculine beliefs were negatively associated with empathy for female violence victims (*r* = -.348, *p* < .001; see Table 1).

**Table 1:** *Bivariate correlations*.

	1	2	3	4	5	6	7	8
**1. Type of video game**	1							
**2. Masculine beliefs**	.203*	1						
**3. Participants' gender**	.022	.507**	1					
**4. Identification with the game character**	.073	.197*	.241**	1				
**5. Empathy for female violence victims**	.000	-.348**	-.137	-.005	1			
**6. Violence ratings**	.809**	.038	-.016	.082	.094	1		
**7. Frequency of play**	.155	.236**	.335**	.117	-.018	.078	1	
**8. Participants' age**	-.521**	-.085	.100	.046	-.003	-.249**	-.020	1

*Note*.

*p < .05.

**p < .01.

Type of video game was coded +1 = violent-sexist games, 0 = violent-only games, and -1 = non-violent games.

We conducted a series of one-way between-subjects ANOVAs to test the effects of type of video game played on three dependent variables: (1) identification with the game character, (2) masculine beliefs, and (3) empathy for female violence victims. The first ANOVA found a significant effect for type of video game played on identification with the game character, *F*(2,152) = 6.06, *p* = .003, *η*^2^ = .073. Post-hoc comparisons using Tukey’s Honestly Significant Difference (HSD) test indicated that participants who played a violent game identified with the character more than did participants who played a neutral game (*d* = 0.62, *p* = .003) or a violent-sexist game (*d* = 0.57, *p* = .04). No differences were found between the neutral and the violent-sexist games (*p* = .67).

The second ANOVA found a significant effect for type of video game played on masculine beliefs *F*(2,152) = 3.33, *p* = .038, *η*^2^ = .004. Post-hoc tests indicated that participants who played a violent-sexist game reported higher masculine beliefs than did participants who played a neutral game (*d* = 0.50, *p* = .03). No statistically differences were found between violent-sexist games and violent-only games (*p* = .24) or between violent-only and neutral games (*p* = .57) on masculine beliefs.

The third ANOVA found no significant effect for type of video game played on empathy for female violence victims (*p* = .31). Descriptive statistics by experimental conditions for each dependent variable are reported in Table 2.

**Table 2:** *Means and standard deviations (in parenthesis) by experimental conditions for each of the considered dependent variables*.

	Neutral game (males *n* = 21; females *n* = 29)	Violent-only game (males *n* = 25; females *n* = 30)	Violent-sexist game (males *n* = 22; females *n* = 26)
**Identification with the game character**	3.89 (1.57)_b_	4.76 (1.21)_a_	4.12 (1.18)_b_
**Masculine beliefs**	2.62 (0.72)_b_	2.77 (0.73)_b_	3.02 (0.86)_a_
**Empathy for female violence victims**	5.03 (0.95)_a_	5.29 (0.92)_a_	5.02 (1.16)_a_

*Note*. Different letters indicate means statistically differences between experimental conditions.

To test our predictions, we then conducted a conditional process model by using the PROCESS macro Model 11 for SPSS with 1000 bootstrapping samples [24]. In this model, the type of video game played was entered as predictor, the identification with the game character as a moderator, masculine beliefs as the mediator, and empathy toward female violence victims as the outcome variable. We predicted that participants’ gender would moderate the effects of the identification with the game character on the relationship between the type of video game and masculinity beliefs. (see Fig 1). Participant age, video game violence rating, and frequency of video game play were also included as covariates.

Considering the moderated path from the type of video game to masculine beliefs, analyses revealed that the main effects of type of video game played, identification with the game character, participant gender, participant age, and frequency of gameplay were not significant (*b*_*s*_ < .56, *t*_*s*_(140)<1.47, *p*_*s*_>.14), whereas the main effect of violence rating was significant (*b* = -.10, *SE* = .047, *t*(140) = -2.07, *p* = .039). The two-way interaction between type of video game played and participant gender was significant (*b* = -1.05, *SE* = .45, *t*(140) = -2.29, *p* = .023), whereas the interactions between type of video game played and identification with the game character (*b* = .02, *SE* = .065, *t*(140) = .32, *p* = .74) and between participant gender and identification with the game character (*b* = -.03, *SE* = .083, *t*(140) = .36, *p* = .71) were both nonsignificant. Crucially, all these effects were qualified by our predicted 3-way interaction between type of video game played, participant gender, and identification with the game character on masculine beliefs (*b* = .26, *SE* = .10, *t*(140) = 2.55, *p* = .011; see Table 3).

**Table 3:** *Regressions of type of video game (neutral*, *only-violent*, *sexist-violent) on empathy for female violence victims when masculine beliefs is the mediator and participants’ gender and identification with the game character are the moderators*.

	*b*	*SE*	*p*
**Masculine beliefs**			
Type of video game	.229	.273	.40
Participants’ gender	.561	.38	.14
Identification with the game character	.063	.049	.20
Age	-.013	.052	.80
Violence rating	-.099	.047	.04
Frequency of play	.026	.043	.54
Type of video game X Participants’ gender	-1.05	.457	.02*
Type of video game X Identification with the game character	.021	.065	.74
Participants’ gender X Identification with the game character	.030	.083	.71
Type of video game X Participants’ gender X Identification with the game character	.265	.103	.01*
**Empathy for female violence victims**			
Age	-.039	.079	.61
Violence ratings	.099	.074	.18
Frequency of play	.052	.061	.38
Masculine beliefs	-.465***	.109	.0001***

*Note*.

*b* = unstandardized beta weight

*SE* = standard error

*p < .05

***p < .001.

**Fig 2:**
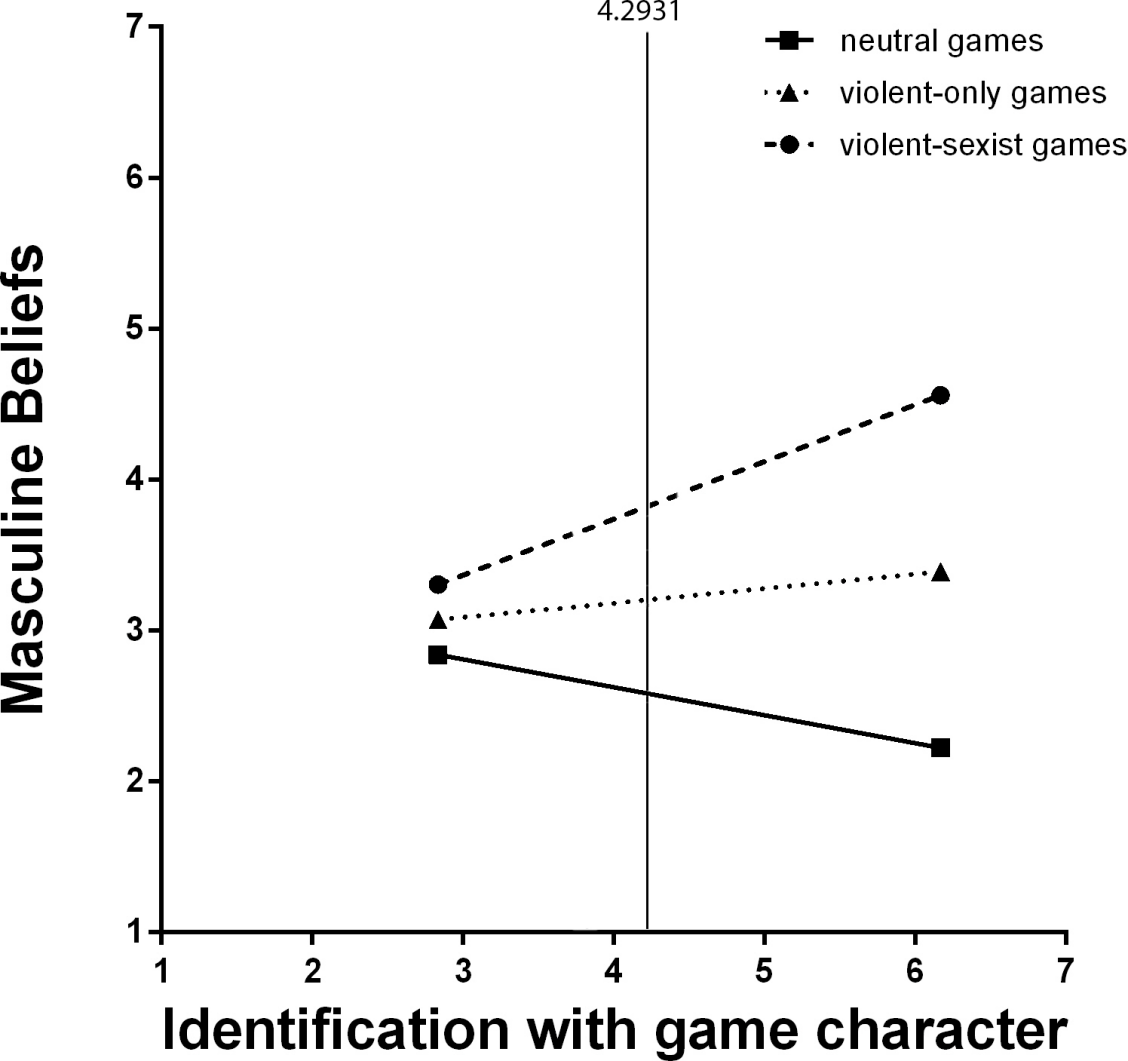
Interactive effect of video game content and identification with the game character on masculine beliefs for male participants. The difference between violent-sexist game players and violent-only and nonviolent game players is significant for values of identification with video game characters greater than 4.2931.

For male participants, simple slope analyses showed a significant positive relationship between identification with the game character and masculine beliefs for males who played with a violent-sexist game (*b* = .32, *SE* = .12, *t*(62) = 2.65, *p* = .009), but not for males who played violent-only game (*b* = .095, *SE* = .078, *t*(62) = 1.21, *p* = .23) or for males who played a nonviolent game (*b* = -.13, *SE* = .97, *t*(62) = -1.33, *p* = .18; see Fig 2). For female participants, there was no significant relationship between identification with the game character and masculine beliefs in any of the three video game conditions (*b*_s_ < .082, *t*_*s*_(76)<1.81, *p*_*s*_*>*.075).

Then, considering the path from masculine beliefs (i.e., the mediator variable) to empathy for female violence victims (i.e., the outcome variable), analyses revealed that masculine beliefs negatively impacted the dependent variable (*b* = -.45, *SE* = .11, *t*(146) = -4.17, *p* < .001).

As first support of our moderated mediational hypothesis, we found a significant indirect effect of the type of video game on empathy for female violence victims via increased masculine beliefs for males [-0.1846 (95% CI = -0.3984 to -0.0410)] but not for females [-0.0406 (95% CI = -0.1318 to 0.0097)] who played with a violent-sexist game. No effect was found for males [-0.0486 (95% CI = -0.1733 to 0.0363)] or females [-0.0294 (95% CI = -0.0799 to 0.0041)] in the violent-only game condition. Similarly, no indirect effect of type of video game on empathy was found for males [-0.0874 (95% CI = -0.0134 to 0.3363)] or females [-0.0181 (95% CI = -0.0783 to 0.286)] in the nonviolent game condition. Further, conditional indirect effects of type of video game on empathy for female violence victims were significant only for males in the violent-sexist game condition at values of identification with the game character greater than 4.2583 [-0.1920 (95% CI = -0.4668 to -0.0492] (see Fig 2). These results suggest that violent-sexist games decreased empathy for female violence victims for boys who strongly identified with the violent game character, and did so by increasing masculine beliefs.

## Discussion

The present research is in line with previous studies showing that violent video games can desensitize individuals to real-life violence [4,31], including violence against women [6]. More important, it moves beyond the question of whether violent games are harmful *per se* to address the important questions of who is most likely to be harmed by violent-sexist video games, and through what mechanism does the harm occur.

A possible answer to the “who” question is players that identify with the violent-sexist game character. Results support the prediction that playing violent-sexist video games increases masculine beliefs and decreases empathy for female violence victims, especially for boys and young men who highly identified with the male game character. Previous research has shown that video games are especially likely to increase aggression among players who identify with violent game characters [11], and that a reduced empathy is one of the major predictor for aggression against women [32]. Exposure to media violence is one of the many factors that can influence empathy levels [33]. Violent video games, in particular, might reduce empathy levels because players are linked to a violent character. If the video game is a first person shooter, players have the same visual perspective as the killer. If the video game is third person, players control the actions of the violent character from a more distant visual perspective. Because they are forced to adopt the visual perspective of the perpetrator, it is difficult for players to put themselves in the shoes of the victim. In general, we argue that it is important to take individual differences into account when considering violent video game effects (see also [34]).

A possible answer to the “why” question (i.e., through which mechanism) is masculine beliefs. Results showed that masculine beliefs were negatively related to empathic feelings for female violence victims. To our knowledge, the present research is the first to elucidate the underlying mechanism that links violent video games playing to desensitization of violence against women.

Speaking to the specificity of our effects, it is noteworthy that type of video game played directly affected identification with the game character and masculine beliefs, whereas it did not directly affect empathy for female violence victims. However, we found an indirect conditional effect of violent-sexist games (vs. only-violent vs. neutral) on empathy, which consistently emerged through the mediation of masculine beliefs and the moderation of identification with the game character. Accordingly, the effects were statistically significant only for highly identified male participants who played the GTA games, which are both violent and sexist. We found no significant effects for violent-only or nonviolent video games on masculine beliefs or empathic feelings. As in previous research (e.g., [8]), gender differences in identification with the game character emerged (i.e., males identified more with the male game character than females did). In addition, males had higher masculine beliefs than females did. This finding can be interpreted in light of the theoretical framework of identification with a virtual character. Video game identification has been considered as an altered experience of the self, in which players may come to perceive themselves to actually be their game character, thus assuming the character’s point of view [13]. This process has been described also as “an imaginative process” involving cognitive, emotional, and motivational dimensions ([35], p. 250). The player shares the character’s perspective (cognitive), feelings (emotional), and goals (motivational) [35,36,37]. Furthermore, identification with a virtual character has been found to be greater in video games with an articulate plot, in which the assigned role fosters a sense of ''vicarious self-perception'' [38]. This is the case with GTA, in which players assume the role of a man who is aggressive, misogynistic, cruel, and greedy. Since that, it is not surprising that we found that male players who identified with the main video game character ended up adopting his point of view more easily than female players, as indicated by an increase in masculine beliefs and a decrease in feelings of empathy for female violence victims.

This investigation of virtual representations of males and females in video games is extremely relevant, because video games have distinct features compared to other forms of media [5] and different effects on males and females. Unlike images in traditional media, game characters are designed to respond to a user’s actions [39], which can promote a powerful experience that goes beyond passive media consumption. Often these interactions mirror communication in the physical world, and users often react to virtual situations in natural and social ways [39,5].

## Limitation and Future Research

Our study, like all studies, has limitations. Few main limitations stand out. First, we used a self-report measure of empathy. Future research should consider other measures of empathy that are less subject to demand characteristics, such as physiological measures (e.g., heart rate, skin conductance).

Second, we examined only two moderators (i.e., participant gender and identification with the game character) and only one mediator (i.e., masculine beliefs). Future research should examine other possible moderators (e.g., trait aggressiveness, social dominance orientation) and mediators (e.g., dehumanization, women objectification; see [22]).

Third, our study was based on a short exposure to violent-sexist video games (i.e., about 25 minutes). Although it is impressive that we were able to obtain significant effects after such a brief exposure, we do not know what the consequences would be for longer exposures. If the effects occur after only 25 minutes of play in a laboratory experiment, they are probably magnified after longer periods of play outside the lab. Indeed, individuals usually play video games for much longer periods of time (from 8 hours to 13 hours per week; [40, 41]). Previous experimental research has shown that the effects of violent video games can accumulate and get larger over time, at least over a three-day period [41].

Fourth, we did not measure sexist thoughts after a certain amount of time, so we cannot ascertain if and how long the observed effects last. However, previous experimental research has shown that the effects of violent video games can last at least 24 hours after gameplay if players ruminate about the content of the game [42,43].

Fifth, *GTA* is a well-known game, and the simple act of playing it could have primed masculine and sexist thoughts regardless of the actual gameplay. Indeed, media audiences often make their assessment of characters and narratives using existing schemes rather than actual on-screen action/content [44]. Thus, future studies should test whether similar effects are obtained for less well-known sexist-violent videogames than *GTA*. Furthermore, future studies should test whether the empathy reduction linked with violent-sexist video games is specific to female violence victims or whether it extends to male violence victims. Future research should also examine video games with female characters that are not depicted in a sexualized manner.

Sixth, we did not actually test whether feelings of empathy mediate aggression against women. As a first step, this study focused on empathy for female violence victims. Future studies should explore whether violent-sexist video games also increase aggression against women.

### Conclusion

One of the best predictors of aggression against girls and women is lack of empathy [32]. The present research shows that violent-sexist video games such as GTA reduce empathy for female violence victims, at least in the short-term. This reduction in empathy partly occurs because video games such as GTA increase masculine beliefs, such as beliefs that “real men” are tough, dominant, and aggressive. Our effects were especially pronounced among male participants who strongly identified with the misogynistic game characters. Daniel Pink was correct in noting that empathy makes the world a better place. Unfortunately, it appears that GTA might make the world a worse place for females.

## Supporting Information

S1 Appendix Pictures used to measure empathy toward a female victim of violence (for illustrative purposes only). Participants indicated how much pain they thought the girl was feeling, and how much they thought she was suffering (1 = *not at all* to 7 = *very much*; Cronbach α = .79).
(DOCX)
