# Supplementary figures and images for: Acting like a Tough Guy: Violent-Sexist Video Games, Identification with Game Characters, Masculine Beliefs, & Empathy for Female Violence Victims
Source: PLoS One. 2016 Apr 13;11(4):e0152121. doi: 10.1371/journal.pone.0152121 (PMC4830454; doi:10.1371/journal.pone.0152121)

**S1 Appendix**


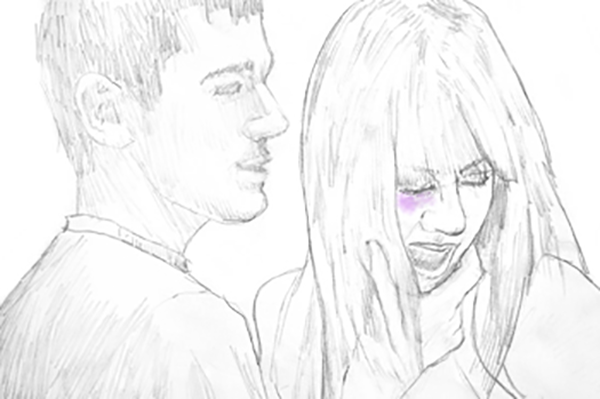


Figure A


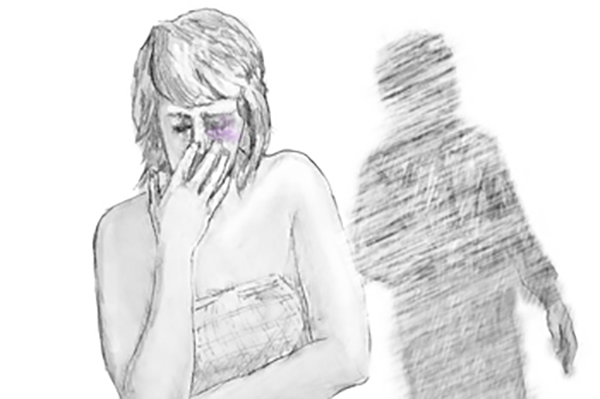


Figure B

Supplement: S1 Appendix — Participants indicated how much pain they thought the girl was feeling, and how much they thought she was suffering (1 = not at all to 7 = very much; Cronbach α = .79). (DOCX) [file pone.0152121.s001.docx]
